# Are phosphodiesterase type 5 inhibitors associated with increased risk of melanoma? A systematic review and meta-analysis

**DOI:** 10.1097/MD.0000000000009601

**Published:** 2018-01-19

**Authors:** Shijian Feng, Liang Zhou, Qinyu Liu, Qing He, Banghua Liao, Xin Wei, Hong Li, Kunjie Wang, Yuchun Zhu

**Affiliations:** Department of Urology, Institute of Urology (Laboratory of Reconstructive Urology), West China Hospital, Sichuan University, Chengdu, Sichuan, People's Republic of China.

**Keywords:** melanoma, phosphodiesterase type 5 inhibitor, risk factor, systematic review

## Abstract

Phosphodiesterase type 5 (PDE5) inhibitors are recommended for patients with erectile dysfunction by American Urological Association and European Association Urology guidelines. However, recent researches have shown that PDE5 inhibitors may lead to increased melanoma risk. Thus, we aimed to explore whether PDE5 inhibitors are associated with increased melanoma risk based on published literatures.

We conducted a systematic online search on PubMed, EMBASE, Cochrane Library, Chinese Biochemical Literature, China National Knowledge Infrastructure, and Chinese Science and Technology Periodical databases to identify the related studies. Odds ratios (ORs), risk ratios, and hazard ratios with 95% confidence intervals (CIs) were extracted and calculated to assess the strength of associations between PDE5 inhibitors and melanoma risk. We also extracted the basal cell carcinoma (BCC) to validate the association in this study.

We included 5 studies containing 100,932 participants in our systematic review and meta-analysis. The calculated results suggested positive results of PDE5 inhibitors on melanoma risk (OR: 1.13; 95%CI: 1.04–1.23). For localized and nonlocalized melanoma, the results were different (OR: 1.22; 95%CI: 1.04–1.43 for localized melanoma) (OR: 0.62; 95%CI: 0.39–0.98 for nonlocalized melanoma). It also showed that PDE5 inhibitors were associated with increased BCC risk (OR: 1.18; 95%CI: 1.11–1.27).

The association between PDE5 inhibitors and melanoma might not be causal due to potential bias (patient selection, and so on) and limitations.

## 1 Introduction

Phosphodiesterase type 5 (PDE5) inhibitors are a kind of drugs which is widely used in clinic. By completely inhibit decomposability of cyclic guanosine monophosphate (cGMP), PDE5 inhibitors are expected to result in relaxation of smooth muscle and maintain penile erection.^[1]^ Thus, it helps erectile dysfunction (ED) patients in maintaining a normal erection, and decreases pulmonary vessels’ pressure in pulmonary vasculature vasodilatation. With only mild complications reported, vision-threatening ocular complications, and hearing loss, PDE5 inhibitor become the first-line therapy for ED.^[1–3]^ Sildenafil, tadalafil, vardenafil, and avanafil are 4 drugs approved by the US Food and Drug Administration specifically for ED treatment.

While scientist focuses on kidney disease and PDE5 inhibitors, because of wide expression of PDE5 in tissues, Arozarena et al^[4]^ declared that sildenafil might induced invasion of melanoma in 2011. Since then, more and more concerns have been raised on this subject. An increased risk of skin melanoma following sildenafil use was also noted by Li et al^[5]^ in 2014 (recent use: hazard ratio [HR], 1.84 95% confidence interval [95%CI]: 1.04–3.22; ever use: HR, 1.92, 95%CI: 1.14–3.22]). Melanoma is the most aggressive type of skin cancers worldwide, especially in western countries with lighter skin colors. It only counts approximately 10% of all kinds of skin cancers, but it causes >80% of skin-related death.^[6]^ A possible explanation is the PDE5 is part of the *RAS-RAF-MEK-ERK* signaling pathway which has been involved in the development of melanoma.^[7]^ Also, the reduced PDE5 expression triggered by activation of *BRAF* gene activation would increase the invasiveness and metastatic potential of melanoma cells,^[4]^ which was noted in >50% melanomas and stimulates melanoma cell invasion and metastasis.^[4,8,9]^ In addition, a recent published study showed sildenafil promotes melanoma growth by potentiating a cGMP-dependent pathway.^[10]^ It is thus plausible that direct pharmacological inhibition of a PDE5 activator may increase the risk of developing melanoma.

Any increase in malignant melanoma risk that is caused by PDE5 inhibitors would have serious public health implications (e.g., 5%–20% of men are affected by ED).^[11,12]^ Moreover, the patent of sildenafil and other PDE5 inhibitors have expired or are soon going to expire in various countries, which lead to the availability of less costly generic versions and the potential for considerably inflated demand in the near future. Therefore, it is important to figure out if there is an association exists between PD5 inhibitors and melanoma risk. However, since 2014, several studies have been published with conflicting results.^[5,13–16]^ We attempted to investigate the association between PDE5 inhibitor use and melanoma risk with a meta-analysis. We also extracted the odds ratios (OR) and HR of PDE5 inhibitor use and basal cell carcinoma (BCC) to re-evaluate the validity of the association of PDE5 inhibitors and melanoma risk.

## 2 Methods

### 2.1 Search strategy and study selection

Two independent investigators, conducted a systematic search of Pubmed, EMBASE, Cochrane Library, Chinese Biochemical Literature (CBL), China National Knowledge Infrastructure (CNKI) and Chinese Science and technology Periodical (CSTP) databases to identify studies related to the association of PDE5 inhibitors and melanoma risk. Search terms were “Melanoma,” “Malignant melanoma,” “Phosphodiesterase type 5 inhibitor,” “PDE5,” “Sildenafil,” “Tadanafil,” “Vardenafil,” and “avanafil.” The references of included studies were also checked manually, in case of missing relevant studies. No language restrictions were applied in this meta-analysis, and the latest online search was in January 2017. The ethical approval of the present study is not necessary, because this is a meta-analysis which is based on published literatures. And no new human participants are involved in this study.

The inclusion criteria were: studies related to the associations of PDE5 inhibitors and melanoma risk; randomized-controlled trials, cohort studies, or case–control studies; studies provided with OR with 95% CI and risk ratios (RR) with 95%CI or HR with 95% CI. Accordingly, case reports, abstracts, conference proceedings, reviews, letters, or repeated publications were excluded. We did not select the ethnicity of the study population. Studies identification, quality assessment, and data extraction were conducted by 2 individual reviewers. If any disagreement appears, a third reviewer was asked to help solving it. All the related articles were retrieved on the internet. If not available, we tried to contact the author directly for full articles.

### 2.2 Quality assessment and data extraction

The quality of each included study was evaluated using the Grades of Recommendation Assessment and Development and Evaluation (GRADE) approach.^[17]^ For nonrandomized controlled studies, we used the Newcastle–Ottawa scale (NOS) to assess the quality of the studies.^[18]^ Studies with a score >7 were considered to be highly qualified.

The basic information of included studies was extracted: name of the first author, year of study recruitment, age range, country, study design, study population data source, PDE5 inhibitor data source, number of patients, and outcome evaluation. Multivariable adjusted ORs, RRs, HRs, and their 95%CIs were also extracted.

### 2.3 Statistical methods

The extracted data from included studies were unadjusted ORs/RRs/HRs with 95%CI except a few multivariable adjusted ORs/RRs/HRs. If the OR was not available nor given the original data, but presented with HR, we used the HR to represent RR. And RR can be transferred to OR using Willi's methods.^[19]^ The pooled out unadjusted risk estimate were used in determining the strength of the association between PDE5 inhibitors and melanoma risk. We also extracted the ORs/RRs/HRs of PDE5 inhibitor use and BCC risk to validate of the association of PDE5 inhibitors and melanoma risk (the risk of BCC was not expected to be associated with PDE5 inhibitors). The heterogeneity test was performed using the χ^2^ test based on *Q*-test and *I*^2^-test.^[20]^ We used random-effects model throughout whole study since there is considerable heterogeneity in some meta-analyses (and where heterogeneity is low, the pooled CI from random model will be the same as that from fixed-effects), regardless of *P* value and *I*^2^. When 2-sided *P* <.05, we defined it as statistically different. Inverted funnel plot visual inspection was used to rate the publication bias among included studies. Besides that, we also performed a sensitivity analysis by excluding each study one by one and recalculate data of the remaining studies. All statistical analyses were performed by Revman software (Version 5.3; Cochrane 13.0; StataCorp, College Station, TX).

## 3 Results

### 3.1 Literature search and characteristics of included studies

**Figure 1:**
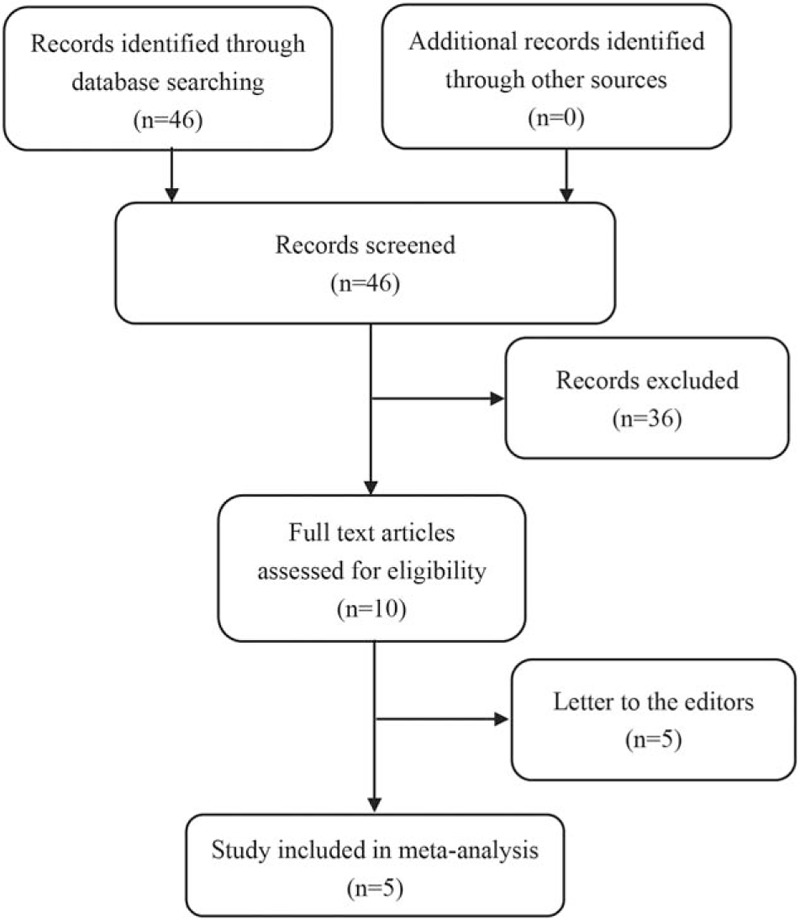
Flow diagram of study selection of meta-analysis.

Our online systematic search identified 46 related studies using the search strategies described above. After screening the titles and abstracts of all studies, we excluded 36 irrelevant studies and 5 letters to the editors. In all, we got 5 studies qualified for our meta-analysis. A flow diagram of study selection was shown to present how we identify pertinent studies (Fig. 1).

The included studies were published between 2014 and 2016, containing 100,932 participants (each ranging from 24,390 to 706,037). All studies were conducted in western, developed countries (USA, Canada, and Denmark). Three studies were cohort studies using HR, and the remaining 2 studies were case–control studies using OR. Only 1 study reported data from 2 data sources, while the other 4 studies reported results of 1 data source. The outcome evaluations of the studies are melanoma, BCC, and squamous cell carcinoma (SCC). All included studies were considered as high-quality studies via NOS evaluation. Sensitivity test did not show abnormal findings. All the summarized characteristics of the included studies are shown in Table 1. Regarding publication bias, the inverted funnel plot visual inspection showed no obvious publication bias.

**Table 1:**
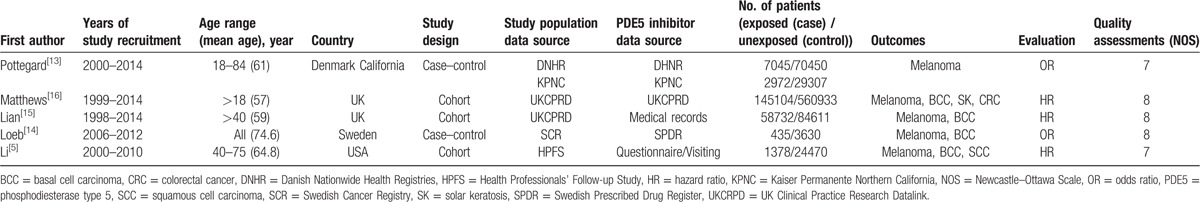
Main characteristics and quality assessments of included studies.

### 3.2 Melanoma

**Figure 2:**
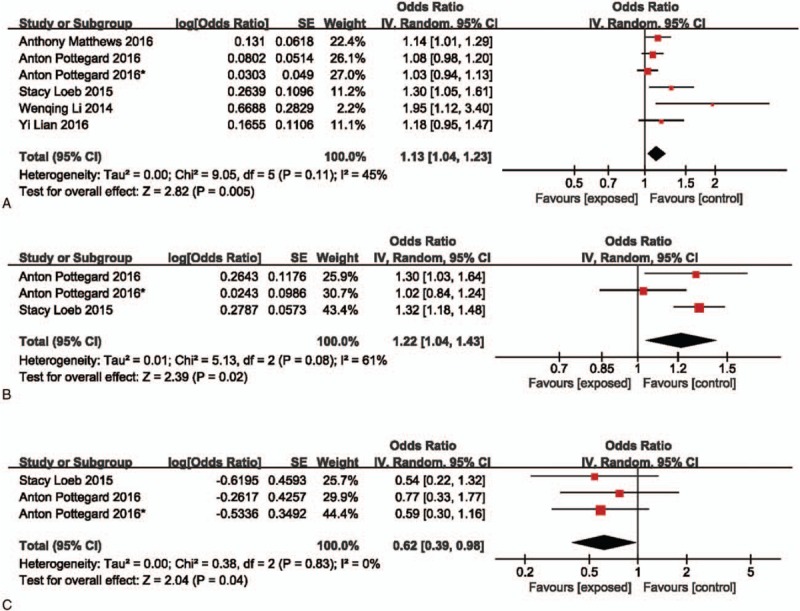
Forest plot of PDE5 inhibitor and melanoma/localized melanoma/nonlocalized melanoma risk. PDE5 = phosphodiesterase type 5.

The association between PDE5 inhibitors and melanoma risk was assessed in all 5 studies. The risk estimate showed a slightly increased risk of melanoma with PDE5 inhibitor use (OR: 1.13; 95%CI: 1.04–1.23). No statistically heterogeneity was noticed among 5 studies (*I*^2^ = 45%, *P* = .11), using a randomized model. (Fig. 2A.)

We also divided melanoma into subgroups to investigate whether there was a difference between localized and nonlocalized melanoma. Localized melanoma was defined as N0 and M0. Nonlocalized melanoma was defined as N1 or M1. Only 2 studies contained required data for localized melanoma. The meta-analysis showed that there was an increased risk of localized melanoma with PDE5 inhibitors (OR: 1.22; 95%CI: 1.04–1.43). The heterogeneity was *I*^2^ = 61%, *P* = .08. For nonlocalized melanoma, the result of 3 databases showed a decreased risk with PDE5 inhibitors (OR: 0.62; 95%CI: 0.39–0.98), while no statistically significant heterogeneity found (*I*^2^ = 0%, *P* = .83) (Fig. 2B and C).

### 3.3 Basal cell carcinoma

**Figure 3:**
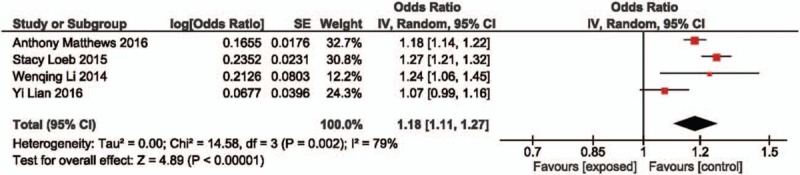
Forest plot of PDE5 inhibitor and basal cell carcinoma risk. PDE5 = phosphodiesterase type 5.

Only 4 studies evaluated the association between BCC risk and PDE5 inhibitors. This meta-analysis showed that PDE5 inhibitors were also associated with elevated risk of BCC (OR: 1.18; 95%CI: 1.11–1.27). However, a statistically noticeable heterogeneity was found (*I*^2^ = 79%, *P* = .002) (Fig. 3).

## 4 Discussion

To the best of our knowledge, this is the first meta-analysis regarding the association between PDE5 inhibitors and melanoma risk. We included 5 studies containing 100,932 participants in this systematic review and meta-analysis. And, we found that PDE5 inhibitor use might associate with increased risk of melanoma, especially with localized melanoma and BCC, while decreased risk of nonlocalized melanoma.

The present studies indicated that there is an association between PDE5 inhibitors and melanoma risk. However, this association must be carefully interpreted, because it is not likely to be causal. Though 4 out of 5 studies used medical record as the definition of PDE5 inhibitor exposure which are highly reliable, all 5 included studies have limitations of one or another kind. In Li et al's^[5]^ study, the exposure was assessed using questionnaires when starting the cohort in 2000, and update on exposure data was not obtained, and lacked important data on timing, duration, and dosing of PDEIs, tumor stage, and use of PDE inhibitors other than sildenafil. Loeb et al's^[14]^ study was limited to patients who had filled with a single prescription, and the proportion of younger men is lower compared to other studies which resulted in marginally lower power and wider confidence intervals for this subgroup. Lian et al^[15]^ using CPRD records prescriptions written by general practitioners and not those filled by patients, leading to some exposure misclassification. Matthews et al's^[16]^ study limitation is they did not have individual-level data on sun exposure, so no directly control for this in the primary analysis. Pottegard et al^[13]^ did not evaluate the BCC or SCC which as negative control of melanoma risk. Moreover, 4 out of 5 studies claimed the association might not be causal. All the studies reported above did not show the participation rate, thus, we do not know whether the participants can represent the general population which is quite important. Though there is no existing evidence on connection between PDE5 inhibitors and risk of BCC, the present study gave out a positive outcome. Moreover, we noticed that in Mathews et al's^[16]^ study, a similar increased risk was also noted in solar keratosis (HR: 1.21; 95%CI: 1.17–1.25). They thought the possible explanation may lies in the sun exposure. Their study showed strong evidence that PDE5 inhibitor users are more likely to have solar keratosis before PDE5 inhibitors prescription, which proposed the possibility that those PDE5 inhibitor users might had experienced excess sun exposure and it is more likely to develop melanoma. Another evidence is that the increased risk was not recorded in the same study for colorectal cancer (HR: 0.91; 95%CI: 0.85–0.98), which is not related to sun exposure. We all know that the common connection among melanoma, BCC, and solar keratosis is sun exposure. However, most included studies, in the current analysis, failed to evaluate sun exposure among participants or the evaluation was not reliable. It also should be addressed that in several studies, the education level and the income of 2 groups were statistically different. This might lead to different life-style between the 2 groups. Thus, the result may be affected by other values which were not be evaluated.

When we compared the results between cohort studies and case–control studies, the overall results are still not consistent (2 positive results in 3 cohort studies, while 1 positive in 2 case–control studies). The most recent cohort studies are conducted by Lian et al^[15]^ and Matthews et al,^[16]^ though Matthews et al came up with positive result, the conclusions of both studies indicated that the association between PDE5 inhibitors and melanoma is not causal (due to sun exposure).

The subgroup analysis suggested different results between localized and nonlocalized melanoma. We considered that may be compatible with a detection bias stemming from that PDE5 inhibitor users had more intensive contact with the healthcare system. The possibility of detection bias is also supported by the observed attenuation of associations after adjustment for education and ambulatory visits in Pottegard et al's^[13]^ study, incorporated in the analyses as markers of healthcare-seeking behavior.

Excepted the results reported above, we also found some interesting results regarding other aspects. Nevertheless, these data cannot be combined due to lack of studies. There are 2 studies reported the PDE5 inhibitors use and risk of SCC. Both of the studies demonstrated no associations (Li et al^[5]^ HR: 0.84l; 95%CI: 0.59–1.20; Lian et al^[15]^ HR: 1.12, 95%CI: 0.87–1.44). SCC is also mechanically not associated with PDE5 inhibitors. Interestingly, 2 studies provided different outcomes of PDE5 inhibitors and melanoma risk. Lian et al's^[15]^ study showed no association, but Li et al's^[5]^ showed positive one. Since Li et al's^[5]^ study only included 14 melanoma patients used sildenafil which is less than Lian et al's^[15]^ study, this difference might be caused by potential bias and mislead the interpretation of the results.

Some studies assessed whether the dosage of PDE5 inhibitors would change the results. Pottegard et al's^[13]^ study contained 2 large databases and showed no statistically different in high use (>100 tablets) and ever use (OR: 0.95, 95%CI: 0.78–1.14; OR: 1.22, 95%CI: 0.99–1.49). Regarding to lower dose, no statistically increased melanoma risk was found. However, in Lian et al's^[15]^ studies showed an increased risk among participants who had received ≥7 prescriptions or more than 25 tablets (HR: 1.3, 95%CI: 1.01–1.69; HR: 1.34, 95%CI: 1.04–1.72). On the other hand, in Lian et al's^[15]^ study, it seems PDE5 inhibitors will not increase or decrease the melanoma risk in fewer prescriptions (1–6) and lower dosage (below 25 tablets) (HR: 1.07, 95%CI: 0.82–1.41; HR: 1.00, 95%CI: 0.75–1.32). These results cannot be combined due to obviously heterogeneity. But considering its study design (Pottegard et al's^[13]^ study was a case–control study, while Lian et al's^[15]^ was a cohort study), the cohort studies provide evidences more powerful than case–control studies. Thus, it is hard to declare that there does exist a dose-dependent association between melanoma risk and PDE5 inhibitors. Also, the proportion of people diagnosed with melanoma differs from countries. According to the included literatures, the highest prevalence of melanoma lies in Loeb et al's^[14]^ study, which is 16.7% of the whole population, while the lowest is only 0.2% in Matthews study. So, this also could be the reason why these results are not consistent.

Regarding PDE5 inhibitors of different types, namly sildenafil, tadalafil and vardenafil, different results were shown in Porttegard et al's^[13]^ study. Usage of 200 to 499 tablets of tadalafil resulted in an OR is 2.05 (95%CI: 1.10–3.84), while the ORs were 1.44 (95%CI: 0.99–2.11) and 1.39 (95%CI: 0.58–3.32) for 200 to 499 and 500+ tablets of sildenafil use in the Danish Nationwide Health Registries (DNHR) database. In Kaiser Permanente Northern California (KPNC) population, the use of 500+ tablets of sildenafil was associated with an OR of 2.50 (95%CI: 0.91–6.99), and the use of 200 to 499 tablets of vardenafil suggested an OR of 1.38 (95%CI: 0.88–2.16).^[13]^ Loeb et al^[14]^ reported that the risk estimates were similar among sildenafil, vardenafil and tadalafil. These results indicated that there might be no statistical difference between different PDE5 inhibitors.^[14]^ Giving the preceding controversies, more high-quality studies are still required for further validation.

There are several potential limitations in the present study. First, there are only 5 studies included in this meta-analysis. Though, the studies are all well designed, more studies are still needed especially when potential bias occurs. The case–control studies and cohort studies might bring potential biases when missing some matches of important values.

Second, other confounding risk factors which are relatively critical to melanoma, such as sun exposure, have not been well evaluated. Some studies reported adjusted results, but vary from each other (e.g., Li et al^[5]^ adjusted for age, body mass index, smoking, physical activity, childhood reaction to sun, number of sunburns, mole count, hair color, family history of melanoma, and sun exposure; Pottegard et al^[13]^ adjusted for use of oral drugs like steroids, weak/moderate topical steroids, and diagnoses of nonmelanoma skin cancer, type 1 or type 2 diabetes, chronic obstructive pulmonary disease, alcohol-related disease, and moderate to severe renal disease, highest education achieved and socioeconomic level; Loeb et al^[14]^ adjusted for Charlson comorbidity index, marital status, and educational level; Lian et al^[15]^ adjusted for age, year of cohort entry, alcohol-related disorders, smoking status, body mass index, precancerous skin lesions, presence of naevi, immunosuppression, use of anti-parkinsonian drugs, Charlson comorbidity score, number of different drug classes used, and number of physician visits in the year before cohort entry, and health-seeking-related variables and disposable income; Matthews et al^[16]^ adjusted for the following number of consultations in year before index date, body mass index, alcohol use, current drinker, ex-drinker, smoking status, current smoker, and ex-smoker.). Due to these variances between studies, we should carefully interpret the results and should consider it as potential limitation. In addition, the details of melanoma were not presented in every study, which made it difficult to ascertain the results of different stages etc.

Third, the majority of participants were western citizens. There are also a great amount of people who use PDE5 inhibitors in developing countries, such as China. But no study has ever explored the association in Asian or African population. Thus, the conclusion of the present study may be not applicable for other races or regions. All of the above factors may possibly affect the association strength observed in our study.

## 5 Conclusion

The results of the present analysis indicated that the association between PDE5 inhibitors and melanoma risk might not be causal. As for subgroup analysis, the local melanoma risk was elevated, while the nonlocalized melanoma was reduced. BCC risk was also increased, which indicated the association might not be legit. Thus, further prospective designed high-quality studies are still required to assess the association between PDE5 inhibitors and melanoma risk.
